# Navigating triple demands: work-life balance challenges and coping strategies among Chinese university teachers pursuing further education

**DOI:** 10.3389/fpsyg.2026.1725160

**Published:** 2026-03-10

**Authors:** Meixin Wu, Chenze Wu

**Affiliations:** Department of Education and Psychology, Hong Kong Baptist University, Hong Kong, Hong Kong SAR, China

**Keywords:** challenges, coping strategies, further education, university teachers, work-life balance

## Abstract

As higher education institutions increasingly expect university teachers to pursue further education, the complexity of balancing professional responsibilities, personal life, and academic advancement has become a pressing concern. However, the lived experiences and challenges faced by these educators remain underexplored. Guided by the Job Demands-Resources (JD-R) Model, this exploratory qualitative study investigated the challenges faced by teachers from diverse types of Chinese universities in balancing these triple demands, as well as the coping strategies that can be used to address these difficulties. Data was collected through semi-structured interviews and analyzed using thematic analysis. Findings revealed a variety of challenges, including workplace constraints, strains in personal affairs, insufficient institutional support, and threats to psychological well-being. Participants also suggested a range of coping mechanisms, such as time and workload management techniques, the importance of supportive institutional policies, interpersonal and family assistance, and practices for maintaining mental health. By applying the JD-R model, this study provides valuable insights into the complexities and difficulties experienced by university teachers and highlights actionable approaches for both individuals and institutions. It informs recommendations for policy refinement and practical support, ultimately contributing to the sustainable development of teacher education and the well-being of educators as they pursue continuing education.

## 1 Introduction

In the contemporary rapidly evolving knowledge-based economy, the importance of lifelong learning and ongoing education for both professional growth and personal enrichment has been widely acknowledged (Mustafa and Lleshi, 2024). University educators, as central figures in driving innovation and generating new knowledge, are increasingly called upon to continually advance their skills and expertise in order to improve the standards of higher education and support broader societal development. With academic requirements and expectations on the rise within universities, many faculty members are seeking additional educational opportunities while continuing to fulfill their regular teaching duties. This pattern emphasizes the vital influence teachers have in meeting shifting educational needs and promoting continuous academic advancement. In this study, further/continuing education refers specifically to doctoral studies (Ph. D., Ed. D., or other professional doctorates) undertaken by in-service university teachers who retain their academic appointments. Such programs are pursued with the aim of deepening disciplinary knowledge, enhancing research capabilities, and improving teaching proficiency, as well as fulfilling institutional or policy requirements for faculty promotion (Kowalczuk-Walędziak et al., 2017; Van Doorsselaere, 2024). This definition is limited to degree-seeking programs and explicitly excludes non-degree professional development activities such as certificates, short courses, and visiting scholar placements.

However, integrating further education into their professional and personal lives poses significant challenges, particularly for university teachers who tend to tackle a complex array of professional obligations. These duties encompass course design, material preparation, assessment development, lecture delivery, and interacting with students (Martin et al., 2019). In addition, faculty members are also required to produce research publications, participate in academic conferences, and secure research funding (Buckley, 2022), often while fulfilling administrative responsibilities (Chen et al., 2022). This multifaceted workload requires substantial investment of time and energy and is likely to frequently extend working hours of faculty members and intensify workplace pressures. The cumulative demands of these roles can adversely impact work-life balance and personal well-being (Zhao P. et al., 2024), consequently leading to heightened stress, diminished work satisfaction, and potentially reduced instructional performance (Jentsch et al., 2023). When educators strive to balance these multiple responsibilities, they may experience heightened risk of burnout, which not only affects their professional performance but may also threaten the long-term sustainability of their careers.

Although further education has the potential to enhance teachers’ professional expertise and self-efficacy (Wasserman and Maymon, 2017), university educators in China frequently encounter considerable barriers that can undermine the benefits of these programs and exacerbate psychological stress. These challenges may be further complicated by institutional policies, limited access to resources, and societal pressures surrounding academic achievement. Considering these concerns, Hu (2024) called for empirical studies to scrutinize how university teachers in China cope with the complexities of balancing professional, personal, and academic development while pursuing continuing education. Therefore, the present study investigated the experiences of Chinese university teachers who are either contemplating or currently pursuing further education, with the aim of identifying the specific work-life balance difficulties they encounter and proposing coping mechanisms. Specifically, the exploratory qualitative research addressed these two research questions.

*RQ1*: What challenges do Chinese university teachers encounter in balancing the demands of work, personal life, and further education?

*RQ2*: What support strategies at the individual and institutional levels can be used to help university teachers balance these competing demands?

By illuminating the unique experiences of university teachers engaged in continuing education, this study provides actionable recommendations for university administrators to strengthen support systems for educators. Furthermore, the findings hold significant implications for policymakers in optimizing continuing education policies and enhancing teachers’ professional satisfaction and overall well-being.

## 2 Literature review

### 2.1 Further education and professional development of university faculty

Further education is widely recognized as essential for university teachers, as it could develop their professional competencies and equip them with the essential abilities to meet the demands of the ongoing education reforms (Wang et al., 2018). This development includes not only formal activities, such as accredited courses and organized workshops, but also informal avenues like independent study, peer collaboration, and similar methods. Such diverse learning opportunities are intended to improve instructional quality, modernize teaching strategies, and foster cooperative engagement among faculty members (Owens et al., 2018). In the context of rapid educational and technological development, it is crucial for educators to keep pace with evolving demands (Wasserman and Maymon, 2017). Previous studies have suggested that participation in further education strengthens research capabilities and fosters teaching innovation among university faculty (Sernaqué et al., 2023; Sernaqué et al., 2023). Such programs not only support the acquisition of new teaching strategies and specialized knowledge but also contribute to improved teaching outcomes (Zhang, 2022), increased job satisfaction, and heightened confidence in managing work-related challenges (Jian, 2023).

In China, the imperative for continued professional development has grown in response to educational internationalization and curriculum reform (Wang et al., 2018). As a result, many higher education institutions now regard engagement in further education as integral to career progression (Bhaskar and Dayalan, 2021). Nevertheless, significant practical obstacles remain, particularly regarding disparities in policy support and resource allocation, which can impede equitable access to professional development opportunities.

### 2.2 Work-life balance of university teachers

Work-life balance refers to the effective management of professional and personal demands to minimize potential conflicts between these domains (Wei, 2023). Achieving work-life balance is closely associated with overall well-being, encompassing aspects such as mental health, job satisfaction, and personal fulfillment (Yang et al., 2018; Yusuf et al., 2020). Within the academic sector, the importance of work-life balance is increasingly emphasized, particularly as ongoing professional development becomes fundamental for educators (Mustafa and Lleshi, 2024). However, the potential tension between engaging in further education and maintaining work-life balance remains a subject of scholarly debate dating back to the mid-20th century (Wyland et al., 2013).

Some scholars conceptualize work-life balance primarily as the balance between professional obligations and family responsibilities (Pan and Sun, 2022; Mayya et al., 2021), while others argue that leisure and educational pursuits should also be considered (Wei C. et al., 2025). With the growing demand for further education, many educators report salient difficulties in reconciling professional development pursuits with their personal lives (Ye, 2022). Recent research has shown that educators encountered various challenges, such as time constraints and limited institutional support, which can hinder teachers’ ability to maintain work-life balance during periods of further study (Ai, 2016; Wang, 2023). In the absence of supportive policies, including flexible work schedules, educators are often compelled to develop their own coping mechanisms (Ozoemena et al., 2021). Despite these challenges, many teachers remain committed to further education as a means of pursuing professional growth and personal satisfaction (Bahramnezhad and Keshmiri, 2025). Some researchers recommend that educators carefully assess their capacity to sustain work-life balance when considering participation in further education (Ozoemena et al., 2021).

### 2.3 Strategies for maintaining work-life balance and pursuing further education

Small but increasing research has begun to identify strategies that can assist university teachers in balancing the demands of work, personal life, and further education. Effective time management emerges as a central concern. Ozoemena et al. (2021) suggest that educators can improve their work-life balance through implementing effective time management strategies, clearly separating their professional and personal lives, and prioritizing self-care. Similarly, Wang (2023) highlights the necessity for strategic planning and prioritization to allocate time effectively across varied responsibilities. Regular physical activity, hobbies, and relaxation methods have been shown to reduce stress and contribute positively to overall well-being (Ozoemena et al., 2021).

However, relying solely on individual coping mechanisms may often be inadequate, and institutional support plays a crucial role as well. Provisions such as flexible work arrangements and more equitable workload distribution can alleviate pressure on university teachers (Wang, 2023). Reductions in administrative burdens, as advocated by Ozoemena et al. (2021), can further enable educators to devote more time to teaching and their own professional development.

Overall, most existing literature tends to emphasize general recommendations, with relatively little attention paid to the unique experiences of individual university faculty managing the combined demands of work, personal life, and continuing education. Therefore, there is an urgent need for qualitative research to examine the individual and institutional contexts of Chinese higher education to better identify effective coping mechanisms. It can enrich current understanding of work-life balance among Chinese university teachers and inform both institutional policy and broader strategies for enhancing faculty development and well-being.

### 2.4 Job demands-resources model

The Job Demands-Resources (JD-R) Model, introduced by Bakker and Demerouti (2007), has become a widely recognized framework for examining occupational stress, work engagement, and employee well-being across diverse professions and organizational contexts. At its core, the JD-R Model posits that every occupation has its own specific demands and resources. Job demands refer to the physical, psychological, social, or organizational aspects of work that require sustained effort and are associated with physiological or psychological costs, examples include workload, time pressure, emotional demands, and work-life conflicts (Bakker and Demerouti, 2007). In contrast, job resources are those facets of work that facilitate the achievement of professional goals, offset job demands, and promote personal growth and development, encompassing factors such as social support, self-management, and institutional policies that enable work-life balance.

The JD-R Model accounts for occupational outcomes through two primary mechanisms. Excessive job demands can exhaust employees’ energy and lead to adverse health outcomes, including psychological stress, anxiety, or burnout (Bakker and Demerouti, 2007; Bakker and De Vries, 2021; Schaufeli and Taris, 2014). Conversely, the availability of robust job and personal resources can buffer the impact of job demands, cultivate work engagement and resilience, and help prevent negative psychological consequences (Bakker and Demerouti, 2007). Personal resources such as self-efficacy, optimism, social competence, and effective time-management are widely recognized as essential for enabling individuals to navigate complex work and life challenges more successfully (Galindo-Domínguez and Bezanilla, 2021; Loeb et al., 2016).

In the context of education, the JD-R Model has been employed to elucidate teachers’ professional challenges and assess their well-being (Granziera et al., 2021). Educators tend to encounter substantial job demands, from increased teaching and administrative workloads to emotional labor and mounting expectations for research productivity, all of which frequently intersect with family responsibilities and goals for personal development (Bakker and Bal, 2010). Empirical studies have highlighted that well-formulated institutional policies and individual coping strategies, such as stress management and work-life boundary setting, function as essential resources, mitigating negative outcomes and promoting teacher retention and job satisfaction (Nwoko et al., 2024; Stockinger et al., 2026).

Owing to its ability to integrate various aspects of occupational demands and supports, the JD-R Model serves as a suitable framework for examining the complex demands experienced by Chinese university teachers as they balance professional, personal, and educational commitments. By systematically examining the interactions between demands and resources, this model offers a robust theoretical basis for identifying both the challenges experienced and the support mechanisms that can sustain teacher well-being and professional effectiveness.

## 3 Methodology

A qualitative approach is well-suited for capturing individuals’ rich and context-dependent accounts and unraveling the complexities inherent in the target issues (Creswell, 2014). Therefore, the current study adopted a qualitative research design to explore the nuanced experiences and perceptions of university teachers who are pursuing further education while maintaining work-life balance.

### 3.1 Research context

The study is situated within China’s evolving higher education sector, in which university faculty members are encountering increasingly heightened expectations regarding research productivity, high-quality teaching, and expanding administrative responsibilities (Wang et al., 2018). At the same time, the Chinese government has constantly underscored the need for university teachers to engage in continuing education, especially as part of broader initiatives to enhance professional development and respond to the rapid evolution of educational technologies (Scott et al., 2023). This emphasis on continuing learning, combined with a variety of professional responsibilities and academic expectations, creates a challenging work environment for university teachers.

In the Chinese higher education context, institutional type can be a potential factor shaping the nature and intensity of work-life balance challenges experienced by university teachers pursuing further education (Sun et al., 2023). Science-oriented universities, for instance, typically exert considerable pressure on faculty to produce research published in high-impact journals and position academic output as a key measure of professional success. This publication-driven environment often intensifies stress and heightens the sense of urgency among staff. In contrast, liberal arts universities tend to privilege practical teaching and workforce preparation, with a strong emphasis on pedagogical effectiveness rather than research publication. Faculty in these institutions may experience different kinds of pressures, which stem from expectations around classroom performance and student outcomes. Comprehensive universities, meanwhile, seek to keep a balance between research excellence and teaching quality, which means they expect faculty to fulfil dual aspects that can compound demands on their time and energy.

Therefore, the researchers decided to recruit participants from different types of higher education institutions. By sampling university teachers from a range of institutional contexts, including comprehensive university, science-oriented university, and liberal arts university, this study sought to capture a diverse array of experiences and perspectives related to work-life balance and the pursuit of further education. Furthermore, this approach enhanced the credibility and transferability of the findings since it provided more nuanced insights that can inform institutional policy and professional development programs across a broader spectrum of Chinese higher education.

### 3.2 Sampling and participants

Ethical approval was obtained from the research ethics committee of Hong Kong Baptist University. Before the implementation of the study, all participants provided written informed consent after being briefed on the research purposes and procedures of the study.

To ensure a diverse and information-rich sample, maximum variation sampling, a type of purposive sampling, was employed (Suri, 2011). This approach was selected to capture diversity in demographic and professional characteristics, thereby maximizing the breadth of perspectives relevant to the research questions. A recruitment post providing a clear overview of the study objectives and explicit eligibility criteria was distributed via WeChat Moments, a widely used social media platform in China.

Participants were eligible if they: (1) were currently employed as university teachers in China, (2) possessed at least a master’s degree, and (3) were currently enrolled in a doctoral program (e.g., Ph. D. or Ed. D.). Individuals participating solely in non-degree professional learning activities, such as certificate courses, short-term professional development, or visiting scholar placements, were not eligible for this study. Interested candidates completed a brief screening survey, which collected relevant background information, including gender, marital status, academic position, educational background, program taught, teaching experience, and type of institution.

Nineteen university teachers expressed interest in participating and met all eligibility criteria. To maximize sample diversity and minimize potential selection bias, two researchers independently reviewed the screening data and then collaborated to select the final interviewees. The selection criteria included gender, marital status, academic position, educational background, program taught, type of institution, and years of professional experience. Based on these considerations, twelve participants were purposively chosen to ensure representation across a broad spectrum of backgrounds and experiences. This is helpful to enhance the credibility and transferability of the research.

Table 1 presents detailed participant information. To protect confidentiality, all identifying details were removed, and pseudonyms were assigned.

**Table 1:** Interview participants’ information.

Pseudonym	Gender	Marital status	Academic position	Educational background	Teaching experience	Program taught	Institutional type
Amy	Female	Married	Lecturer	Master’s degree	5 years	English Translation	Comprehensive University
Yoyo	Female	Married	Lecturer	Master’s degree	7 years	College English	Comprehensive University
Ashley	Female	Married	Associate Professor	Master’s degree	17 years	College English	Science-oriented University
Tom	Male	Married	Teaching Assistant	Master’s degree	4 years	Economics	Science-oriented University
Leo	Male	Single	Lecturer	Master’s degree	4 years	Business English	Liberal Arts University
Mei	Female	Married	Assistant professor	Master’s degree	10 years	Law	Science-oriented University
John	Male	Married	Lecturer	Master’s degree	8 years	Economics	Comprehensive University
Simon	Male	Married	Lecturer	Master’s degree	5 years	Business Administration	Comprehensive University
Linda	Female	Married	Lecturer	Master’s degree	9 years	English Translation	Liberal Arts University
Kelly	Female	Single	Teaching Assistant	Master’s degree	2 years	Sociology	Liberal Arts University

### 3.3 Data collection

Semi-structured interviews allowed the researchers to guide the interactions with pre-determined open-ended questions as well as remain the flexibility to pursue emerging topics and unexpected insights in real time (Cohen et al., 2018; Merriam and Tisdell, 2016). Therefore, semi-structured interviews were used to collect qualitative data to elicit a comprehensive and nuanced understanding of participants’ lived experiences with work-life balance and further education. All interviews were conducted individually online, which was convenient and accessible for participants regardless of geographical location or scheduling constraints. After a comprehensive review of relevant literature (Ozoemena et al., 2021; Hu, 2024; Jentsch et al., 2023), an interview guide was developed to holistically explore individual perspectives on the issue. Participants were given the option to respond in either Chinese or English, according to their preference; ultimately, all of them chose to use Chinese, their first language, as it allowed them to express their thoughts and experiences most effectively. All interviews were audio-recorded to ensure accurate recording of their responses for subsequent analysis.

### 3.4 Data analysis

The qualitative data obtained from the interviews were analyzed using the six-step thematic analysis proposed by Braun and Clarke (2006). All interviews were initially transcribed verbatim in Chinese. Each participant was provided with their transcript and invited to review and verify the accuracy and intended meaning of their responses. This process, known as member checking, helped enhance the study’s credibility (McKim, 2023). After participant verification, two researchers collaboratively translated the transcripts into English. Any ambiguities encountered during translation were discussed and resolved to ensure that the original meanings were preserved. To further ensure translation quality and faithfulness to the original meaning, a professional translator subsequently reviewed the English transcripts to verify their accuracy.

Subsequent data analysis was conducted using these validated English transcripts. The two researchers immersed themselves in the interview data by reading the English transcripts multiple times, which ensured an in-depth understanding of the participants’ experiences and perceptions. After familiarization, both researchers independently conducted line-by-line manual coding of each transcript, identifying meaningful text segments and assigning initial codes that reflected participants’ intended meanings. To minimize the risk of meaning loss or misinterpretation due to translation, both researchers referred to the original Chinese transcripts during the coding process. When uncertainties arose regarding the meaning of statements in the English version, the Chinese source text was consulted to ensure accurate understanding and coding. This ensured that interpretations accurately reflected participants’ perspectives as originally expressed in their native language.

Once initial coding was completed, the researchers systematically compared and discussed their codes, collaboratively clustering them into potential themes through constant comparison across transcripts. This process can capture the full breadth of participants’ experiences and perspectives. The constructed codes and themes were then jointly reviewed, refined, and named to ensure their relevance and conceptual clarity. Reflexivity was maintained throughout the analysis by engaging in continuous discussion and collective reflection at each stage of coding and theme generation. These exchanges helped reduce potential biases and enhance the trustworthiness of the study. Any discrepancies or disagreements were addressed through discussion until consensus was achieved.

**Figure 1:**
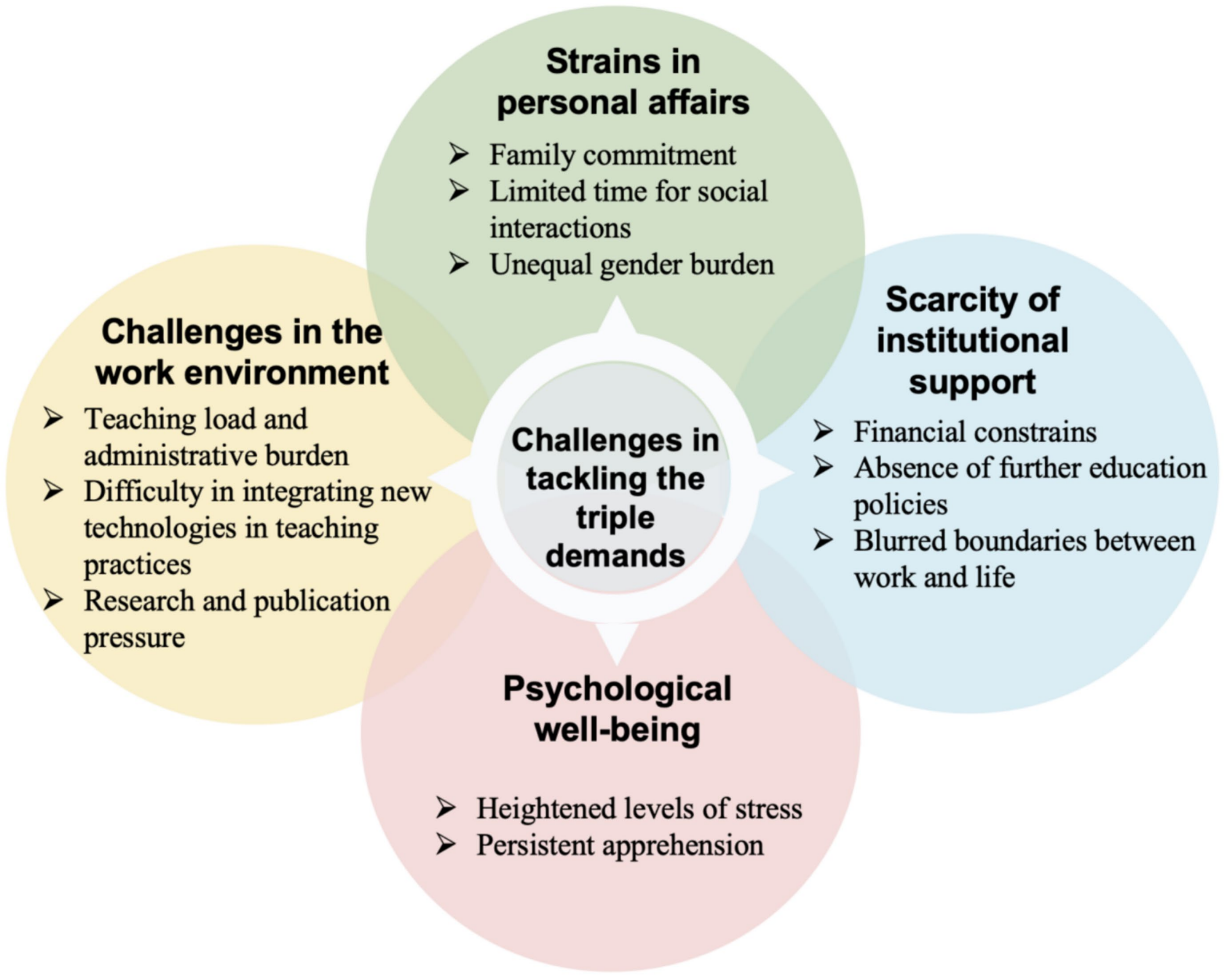
University teachers’ challenges in balancing work, life, and further education.

**Figure 2:**
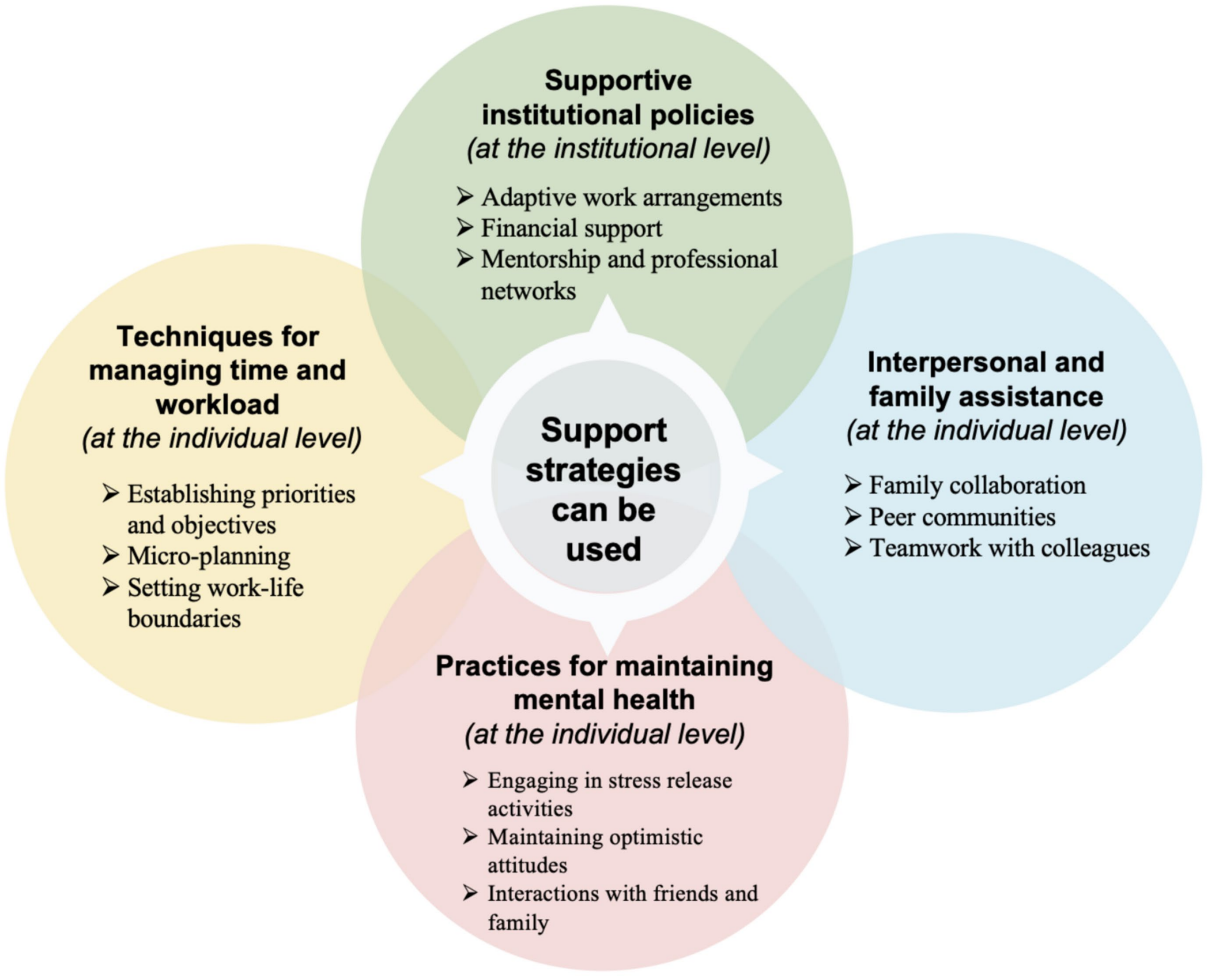
Support strategies to balance work, life, and further education.

Additionally, regular peer debriefing sessions were held with a professor specializing in teacher education, who had no involvement in the data collection process. These sessions focused on reviewing the coding process and emerging themes, and constructive feedback was also sought from the professor to further enhance the trustworthiness of the study. The final themes are presented in Figures 1, 2. It should be noted that the relationships among themes illustrated in these figures are organizational (taxonomic) and do not imply any directionality or causality.

## 4 Findings

### 4.1 Challenges in balancing work, life, and further education

Examination of the interview data revealed four central issues that university educators grapple with as they strive to juggle their career obligations, personal lives, and pursuit of additional qualifications (see Figure 1).

#### 4.1.1 Challenges in the work environment

Participants reported a range of challenges in their work environment. A recurrent theme was the substantial teaching workload and the administrative responsibilities. For example, Kelly described her situation: “*I have a heavy teaching load of almost 16 h per week. Sometimes, apart from this 16-h workload, I also have to teach night classes.*”. Apart from this, many participants, including Tom, illustrated, “*These include attending some communities, giving thematic lectures to students as a homeroom teacher, sharing college recruitment information on social media apps and writing weekly department meeting reports*.” Moreover, participants highlighted the increasing pressure to integrate new technologies in teaching practices, particularly in the context of rapid developments in artificial intelligence (AI). Ashley further elaborated on this challenge, stating that she must “*spend tons of time and effort on learning more than one technology, especially for teachers over 40 years old*,” a demand that significantly encroached upon personal time and complicated the pursuit of further education. Besides, several participants expressed concerns about research and publication pressures.

#### 4.1.2 Strains in personal affairs

Participants reported various personal life strains that could impede their pursuit of further education. Family commitment, containing both childcare and eldercare, was frequently perceived as a major source of pressure. For instance, Linda shared, “*After a full day of teaching, I have to take care of my children and my elderly parents. I rarely have any quiet time for myself, let alone to study*.” This dual care-giving role not only consumed physical energy but also left little room for personal growth or academic advancement. Another prevalent issue was the limited time available for social interactions. Many participants highlighted the sacrifices required to meet work and study commitments. John expressed this tension and stated, “*I often miss important gatherings with my friends because of my workload and study commitments*.” Long-term lack of social activities can lead to social isolation and deterioration of interpersonal relationships, which in turn can cause well-being and health issues.

Unequal gender burden emerged as a distinct and significant focus, with married female teachers particularly affected. Several married female participants pointed out that the responsibility of housework inevitably falls on them. Linda explained, “*Even after work, all the housework and cooking fall on me. My husband helps sometimes, but it is still on my shoulders most of the time*.” Such gendered disparities struck a sympathetic chord with other participants, who linked these additional tasks to increased exhaustion and diminished opportunities for self-development, including further education.

#### 4.1.3 Scarcity of institutional support

Monetary constraint was reported as a notable barrier. Participants indicated that the cost of tuition and other related expenses for further education placed a significant strain on their households. Many participants expressed dissatisfaction with the limited financial assistance their institutions offer for pursuing further education. They pointed out that their only option for pursuing further education is to take unpaid leave of absence, as the university does not provide any financial support. The expense was considerable since they had to “*pay rent for accommodation on campus, no matter whether we live in or not and the social and health care insurance*.”

The absence of further education policies emerged as another commonly reported challenge. Participants expressed concerns regarding the lack of formal policies or resources to assist teachers aiming to pursue further study. Additionally, the boundaries between work and life were frequently described as blurred, especially in the context of further education.

#### 4.1.4 Psychological well-being

Participants consistently reported that pursuing further education alongside demanding work and personal responsibilities has led to heightened levels of stress. Similarly, Kelly described “*After teaching night classes, when I come home, I still have to finish tasks from further education, which makes me break down*.” Moreover, persistent apprehension was also mentioned by participants. John and Leo both noted that a lack of social activities contributed to “*feelings of guilt, social isolation, and deteriorating relationships*,” which enhanced their anxious feelings. Most participants hoped to work hard to “*make trade-offs between work and academics*” in order to “*excel in both fields*.” However, they struggled to realize such a high self-expectation and this consequently resulted in aggravated mental issues, including anxiety.

### 4.2 Support strategies for university teachers to balance work, life and further education

In the semi-structured interviews, participants suggested a wide range of support strategies that can be used to help them adjust and achieve work-life balance while pursuing further education (see Figure 2).

#### 4.2.1 Techniques of managing time and workload (at the individual level)

Establishing priorities and objectives was regarded as an essential strategy for managing the competing demands of work, life, and further education. Participants claimed notable benefits from prioritization and goal-setting and emphasized the practice of distinguishing between essential (“*must-do*”) activities and less urgent (“*can-wait*”) tasks. As Amy reflected, “*I learned to list my daily tasks and identify what truly needs to be done that day. If something can wait, I do not let it stress me out*.” For some this even meant completing only the “*necessary*” teaching tasks to meet basic professional requirements and prioritizing time for further education. Besides, micro-planning strategies were widely adopted, with participants employing digital calendars, mobile apps, and detailed schedules to allocate time for teaching, research, and study. Amy added that setting alarms according to her timetable helped her complete urgent tasks efficiently. These approaches were reported to enhance productivity and reduce feelings of being overwhelmed.

Setting work-life boundaries was recommended as well. For example, Ashley commented, “*I’m gradually withdrawing from some unnecessary communities, and I will not have to attend the community meetings every week anymore, so I can stay focused on my priorities*.” Likewise, Tom remarked, “*I decided not to do administrative work or performance appraisal tasks. I will have more time instead*.” In addition, some participants, like Leo, explicitly communicated their study schedules to colleagues and family, and they found that, in this way, they would have the awareness to avoid disturbing them during those periods, and “*it has really helped me concentrate*.”

#### 4.2.2 Supportive institutional policies (at the institutional level)

Several participants highlighted that supportive institutional policies, such as adaptive work arrangements, financial support, and mentorship and professional networks, are critical to successfully balancing work, life, and further education. Adaptive work arrangements were consistently identified as one of the most needed changes. Some participants discussed the possibility of reduced teaching loads or a remote teaching format as valuable options. All of them mentioned it is significant to reduce some irrelevant workload to teachers, such as administrative tasks. Linda reflected, “*If I could have a decreased course load or flexible teaching schedule, I would be less stressed and focus more on my academic performance for my further education*.” Similarly, Leo commented, “*Teaching students online instead of face-to-face, I could use my time much more efficiently*.”

Financial support from institutions was also considered indispensable. Yoyo suggested, “*Universities should provide paid leave to teachers for further education*.” Amy further mentioned that, when unpaid leave is taken, the university should “*cover the social and health care insurance*” rather than requiring teachers to pay for it themselves. In addition, mentorship and professional networks further assisted participants in managing time and workload effectively. Senior colleagues who had overcome similar challenges were frequently regarded as valuable sources of advice. Many participants felt that “*such support helps me feel less isolated and more confident in balancing competing responsibilities*.”

#### 4.2.3 Interpersonal and family assistance (at the individual level)

Beyond individual effort, achieving work-life balance was also facilitated by accessing support from both family and the social sphere. Family collaboration was considered a valuable strategy, particularly among married female teachers. Participants stated the importance of negotiating shared responsibilities for household and caregiving duties, which allowed greater focus on academic and professional commitments. As Mei explained, “*My husband agreed to help more with the cooking and looking after our children during my exam periods. Without his support, I could not manage my studies*.” Peer communities were also identified as useful in fostering motivation and providing practical guidance in further study. Academic study groups, informal networks, and circles of peers enabled the exchange of advice and encouragement. Yoyo highlighted, “*When I was struggling to finish a paper, my study group reminded me of deadlines and shared their notes. Sometimes just knowing others are in the same boat is reassuring*.” These networks reduced the sense of loneliness that people often experience when they were working and pursuing further education simultaneously.

Teamwork with colleagues further alleviated time pressures. Several participants reported engaging in team-teaching, co-authoring papers, or sharing workloads to support each other’s academic goals. Ashley shared, “*My colleague and I decided to split class preparation for a semester, and we co-authored one of the research papers. Then, we do not have to learn too many new instructional technologies. It saved us both a lot of time*.” Such collaborative practices allowed participants to meet institutional obligations while progressing in their studies.

#### 4.2.4 Practices for maintaining mental health (at the individual level)

Participants highlighted the importance of using strategies to maintain mental health. Engaging in stress release activities was a common approach, with many reporting that regular exercise, meditation, or pursuing hobbies provided essential relief from academic and professional demands. As Kelly described, “*Every morning, I do yoga for thirty minutes before checking my work emails. It helps me start the day with a clear mind and less anxiety*.” Maintaining optimistic attitudes was identified as another crucial practice in building resilience. They noted that this mindset can help sustain their motivation as they navigate the difficulties of managing multiple responsibilities. Several participants contended that the benefit of setting realistic expectations and celebrating small achievements. Mei reflected, “*I remind myself that setbacks are normal and making progress takes time. When I finish a tough assignment or receive positive feedback, I consciously take a moment to appreciate it*.” This emphasis on optimism and self-compassion helped participants maintain motivation despite setbacks or competing demands.

Interactions with friends and family also played a significant role in emotional regulation. Social support was described as essential for managing stress and maintaining well-being. Several participants shared those informal conversations and emotional encouragement from their support networks alleviated their feelings of “*isolation*” or “*burnout*.”

## 5 Discussion

This study offers valuable insights into how university teachers in China navigate the complexities of balancing professional, personal, and academic development while pursuing further education, which makes responses to Hu’s (2024) call for empirical studies to address this issue. In the current competitive academic landscape, the pursuit of higher qualifications is increasingly viewed as a strategic choice for university teachers seeking greater job opportunities and higher salaries. Despite these clear career benefits, participants in this study reported a range of salient challenges inherent in managing these demands, which is consistently highlighted in prior research (Wei and Ye, 2022; Zhang et al., 2024).

The JD-R Model has been increasingly utilized to explore work–family conflict and work-life balance within academic settings (e.g., Sarwar et al., 2021; Bakker et al., 2023). This framework is particularly relevant for understanding the experiences of Chinese university teachers, who are struggling to balance the competing demands of professional responsibilities, personal life, and continuing education. Thus, the JD-R Model can serve as an appropriate theoretical foundation for the present study. Through the lens of the JD-R Model, this study offers nuanced insights into how the interplay between job demands and various resources, personal, social, and institutional, shapes the experiences and coping strategies of Chinese university teachers pursuing further education.

Challenges in the work environment were identified as major obstacles for Chinese university teachers who are pursuing further education. From the JD-R perspective, intensified job demands include heavy teaching and research workloads, extensive administrative responsibilities, and pressure to integrate new technologies. If not offset by sufficient resources, these demands can elevate psychological stress and adversely impact well-being among educators (Bakker and De Vries, 2021). As emphasized by Wei Y. et al. (2025), heavy teaching loads and administrative duties leave them with little time or energy for their own academic advancement. Tasks such as preparing lessons, grading assignments, and handling paperwork often take priority, which might force teachers to sacrifice time that could be spent on their studies. In addition, the pressure to integrate new technologies into teaching is a growing concern (Huang and Zhao, 2025). Teachers are expected to quickly adapt to digital technologies and artificial intelligence tools, which can be especially overwhelming for those with less experience in using technologies and often result in additional work and stress. Moreover, the need to conduct research and publish papers further intensifies the pressure, since teachers are required to meet high scholarly expectations while still fulfilling their regular professional responsibilities (Zhao S. et al., 2024). According to the JD-R framework, the convergence of these factors constitutes high job demands that are prone to drain educators’ energy and impede work-life balance (Hlado and Harvankova, 2024; Sokal et al., 2020). Thus, enhancing job resources, such as reducing administrative tasks, implementing flexible scheduling, and providing targeted technological training, can be instrumental in managing these demands and supporting both well-being and professional development.

Personal life strains emerged as a pivotal challenge for Chinese university teachers balancing the interplay between professional duties and further education (Wei C. et al., 2025). Many participants reported significant family responsibilities, including childcare, eldercare, and household management, which often conflicted with study and work commitments. Notably, unequal gender burdens were especially pronounced among female teachers, who frequently shouldered a disproportionate share of domestic duties alongside their academic and professional roles. This is similar to the findings of Su and Jiang (2023) and Dong et al. (2025). The gendered imbalance not only intensified stress but also contributed to feelings of frustration and inequality. This persistent gender imbalance not only exacerbates stress but also contributes to frustration and feelings of inequity. Within the JD-R framework, personal and family demands exacerbate overall strain (Demerouti and Bakker, 2023), which underscores the necessity for organizational and societal resources to counterbalance these non-work-related pressures.

From a broader perspective, these gendered burdens are shaped by underlying cultural norms and institutional practices. Longstanding societal assumptions cast women as primary caregivers (Zygouri et al., 2021), while the lack of supportive institutional policies, such as accessible childcare, equitable parental leave, and flexible work arrangements, intensifies the unequal distribution of domestic duties (Horta and Tang, 2023). Promotion systems that prioritize continuous productivity further disadvantage women and reinforce gender gaps within academic careers (Moors et al., 2022). Accordingly, addressing these challenges requires coordinated efforts at multiple levels. For example, universities can introduce inclusive and supportive measures, such as extended and equitable parental leave and subsidized childcare. At the policy level, government-led initiatives that promote awareness, encourage shared domestic responsibilities, and support flexible work options are necessary. Furthermore, as underlined by Maguire et al. (2025), offering targeted mentorship and leadership development for female educators can cultivate supportive environments and contribute to closing the gender gap.

Scarcity of institutional support was another important barrier for Chinese university teachers striving to balance career progression, personal life, and further education. Participants frequently highlighted the lack of financial assistance for professional development or further education, which can deter their academic advancement. Beyond monetary constraints, the lack of targeted institutional policies for further education, particularly flexible scheduling and reducing teaching workload, further exacerbates this challenge. The absence of such supportive frameworks often leads to a blurring of boundaries between professional responsibilities and personal aspirations (Wang, 2023). This means teachers are forced to sacrifice leisure and family time to fulfil academic and work demands (Wei C. et al., 2025). As underscored by the JD-R Model, the lack of adequate job resources is likely to increase individuals’ susceptibility to stress and burnout (Bakker and De Vries, 2021). Therefore, in light of these findings, universities should re-evaluate current institutional policy and funding allocations and introduce more responsive support mechanisms to enable faculty to thrive amid the intensifying demands of higher education.

Issues related to psychological well-being, particularly stress and anxiety, were also salient according to participants’ statements. The simultaneous demands of teaching, research, and academic advancement contribute to sustained levels of psychological pressure, and educators often report symptoms of chronic stress, sleep disturbances, and persistent feelings of overwhelm. This ongoing strain might not only impair their emotional well-being but also undermine cognitive functioning and work performance (Jentsch et al., 2023), and consequently, create a cycle that is difficult to break. Furthermore, the lack of institutional mechanisms to support mental health, such as counseling services or stress management programs, further strengthens these challenges. The JD-R Model underscores that persistently high job demands with insufficient resources can lead to adverse health outcomes, such as psychological distress and diminished work engagement (Bakker and De Vries, 2021). Consequently, universities are urged to prioritize mental health by strengthening psychological support systems and ensuring faculty have access to support when needed. Such efforts enhance both the personal and professional resources available to educators and, therefore, make them more resilient in the face of occupational pressures.

It is recommended for university teachers, especially those who are pursuing continuing education, to utilize time and task management strategies. Participants highlighted the critical role of proactive self-regulation in coping with the demands of professional, educational, and personal life. Effective prioritization and goal setting have been highlighted by many scholars (Hudson and Hudson, 2016), since it enables teachers to identify urgent and important tasks and allocate limited resources toward activities most aligned with their immediate objectives and long-term aspirations. Micro-planning should also be valuable for mitigating overwhelm and maintaining momentum. According to Santiago (2023), there are many commonly used micro-planning strategies, including breaking down complex commitments into manageable steps and scheduling specific time blocks for study, teaching, and familial responsibilities. Furthermore, clear boundary setting, like refusing unnecessary work and tasks or sharing study schedules with colleagues and family, was essential for minimizing conflicts and protecting personal well-being. These strategies can not only foster a sense of control but also reduce the mental strain associated with multitasking and multiple obligations. Within the JD-R framework, such self-management techniques can be understood as personal resources that enable teachers to navigate high demands, maintain motivation, and enhance resilience in the face of competing obligations. This aligns with prior research emphasizing the importance of self-management skills in sustaining work-life balance in academic professions (Palanca, 2025).

Supportive institutional policies should also be underscored. For example, universities should consider implementing flexible work arrangements, including adaptable teaching schedules and remote options, to grant educators the autonomy necessary for balancing academic and personal obligations. Such moves could ensure teachers are not overwhelmed with regular workloads while pursuing further education. Furthermore, according to Borres and Paglinawan (2025), the provision of financial support, such as scholarships, travel grants for conference attendance, paid leave, or research funding, should be considered to alleviate monetary pressures that might otherwise deter teachers from furthering their qualifications or balancing multiple roles. Besides, mentorship programs and professional networks offer not only vocational guidance but also psychosocial support. Universities are advised to develop formal mentorship programs that pair junior or mid-career faculty with senior colleagues to foster both academic growth and well-being, as well as establish peer-support groups for sharing resources and coping strategies (Maguire et al., 2025). These forms of institutional support, recognized within the JD-R framework as important job resources (Han et al., 2020), help mitigate the adverse impact of high job demands and simultaneously promote engagement, satisfaction, and retention among university teachers (Wei C. et al., 2025).

Interpersonal and family assistance was another important source for tackling the intertwined demands faced by Chinese university teachers. The study emphasized that spousal understanding, shared domestic responsibilities, and intergenerational care-giving arrangements enabled university teachers to devote necessary time and energy to academic and professional pursuits, which revealed the importance of family collaboration. This aligns with the findings of Umma and Zahana (2021). Peer communities, both within and beyond university settings, provided a vital platform for emotional encouragement, resource sharing, and mutual validation, thereby helping them alleviate the sense of isolation (Wei C. et al., 2025). Further, collaboration with colleagues, such as team teaching or co-authoring a manuscript, contributed to a supportive work environment where challenges could be collectively addressed and successes jointly celebrated. According to Wang (2023), these forms of social and familial support not only mitigated individual stress but also fostered resilience, adaptability, and a sense of belonging. From the perspective of the JD-R Model, social support, whether provided by family, peers, or colleagues, serves jointly as personal and job resources (Kakkar et al., 2024). Such support strengthens individuals’ ability to alleviate stress and fosters positive outcomes, even when they are confronted with considerable job demands.

Moreover, a wide range of practices for maintaining mental health were also recommended. Stress release activities, including physical exercise, meditation, or pursuing hobbies, were widely reported as effective means to temporarily disengage from academic and professional pressures (Wang, 2023), thereby preventing burnout and promoting psychological recovery. Alongside these practical approaches, the cultivation of a positive mindset played a transformative role. Participants emphasized the importance of reframing challenges as opportunities for growth, maintaining optimism in the face of setbacks, and celebrating incremental progress. Furthermore, regular interactions with friends and family, whether through casual conversations, shared meals, or moments of mutual support, provided essential emotional nourishment, fostering a sense of belonging and reinforcing personal motivation. These self-adjustment strategies are corroborated by recent research (e.g., Umma and Zahana, 2021), which underscores the efficacy in enhancing adaptability and long-term job satisfaction among academic professionals.

## 6 Conclusion

This study contributes to the current limited understanding of the work-life balance challenges and their coping strategies. By investigating the persistent barriers and the adaptive strategies employed by participants, the findings emphasize an urgent need for changes in both institutional policy and teaching practice. Collectively, the results advocate for more flexible time management, supportive institutional and financial support, and active self-initiated coping strategies.

### 6.1 Practical implications

The findings of this study underscore practical implications from the aspects of the educational institutions, university teachers, and their family members. For educational institutions, it is imperative to recognize and aim to mitigate teachers’ work-related challenges and further education barriers by implementing more flexible and supportive institutional policies, such as adaptable teaching schedules, reduced administrative burdens, and targeted financial support. Institutions are also recommended to provide robust professional development opportunities, including technology training and mentorship, to ease the pressure of integrating new digital tools and meeting high-demanding research expectations. Additionally, it is crucial to foster a culture that values mental health and well-being, and promotes clear boundaries between work and personal life, which are essential to mitigate identity strain and emotional exhaustion among educators.

From the perspective of teachers, they should develop an awareness of adopting effective time and task management strategies, including prioritization, micro-planning, and boundary-setting, which is helpful to balance their triple roles. Teachers are also suggested to engage in self-adjustment practices, maintain a positive mindset, and participate in stress-relieving activities, since these activities have been found effective in sustaining emotional and mental health (Wang, 2023). Furthermore, cultivating and sustaining a positive mindset should also be a value, which enables teachers to reframe challenges as opportunities for growth and thus reduce their feelings of overwhelm and foster higher adaptability amid shifting academic and professional landscapes. Beyond individual strategies, building robust peer communities and seeking close collaboration with colleagues play a pivotal role in alleviating feelings of isolation and professional strain. They can share coping strategies, provide mutual encouragement, and engage in jointly solving problems, which can not only deepen their personal support networks but also help distribute workloads, facilitate resource sharing, and collectively navigate institutional challenges. Ultimately, their professional efficacy and psychological resilience can be enhanced during the collaborative process (Wei C. et al., 2025).

Additionally, family members also play a critical role in helping teachers achieve balance. They should keep active cooperation and open communication, which enable them to share domestic responsibilities more effectively and support each other’s psychological well-being. Furthermore, the unequal gender burden needs to be noticed and addressed through promoting an equitable distribution of household duties, which is particularly important for supporting female educators.

### 6.2 Limitations and directions for future studies

However, the study has several limitations. First, the study’s relatively small sample size may not adequately represent the full spectrum of experiences and work-life balance challenges encountered by Chinese university teachers pursuing further education. Future studies should consider recruiting larger and more diverse participants to improve the transferability of the findings. Second, the exclusive reliance on semi-structured interviews may possibly restrict the breadth of data collected. To address this issue, future research is advised to implement a mixed-methods design, which could incorporate surveys, focus groups, or observations, to enable data triangulation and strengthen the credibility of the findings. Moreover, future research could explore the long-term impact of work-life balance challenges and coping strategies on educators’ professional development and personal well-being, as the current study’s cross-sectional design does not allow for an assessment of the enduring effects over time. Additionally, although the study identified stress and anxiety as key concerns, it did not explore the potential long-term psychological consequences of these challenges. Future studies could investigate how chronic stress impacts educators’ mental health and career sustainability.

## Data Availability

The original contributions presented in the study are included in the article/supplementary material, further inquiries can be directed to the corresponding author.
