# Correction: Navigating triple demands: work-life balance challenges and coping strategies among Chinese university teachers pursuing further education

**DOI:** 10.3389/fpsyg.2026.1908090

**Published:** 2026-07-03

**Authors:** Meixin Wu, Chenze Wu

**Affiliations:** Department of Education and Psychology, Hong Kong Baptist University, Hong Kong, Hong Kong SAR, China

**Keywords:** challenges, coping strategies, further education, university teachers, work-life balance

Potentially identifiable data was erroneously captured in the published article. This has now been removed and corrected.

The original version of this article has been updated.

